# Local epidemiology and resistance profiles in acute uncomplicated cystitis (AUC) in women: a prospective cohort study in an urban urological ambulatory setting

**DOI:** 10.1186/s12879-017-2789-7

**Published:** 2017-10-16

**Authors:** Michael Seitz, Christian Stief, Raphaela Waidelich

**Affiliations:** 1 UroClinic München GbR – Campus Bogenhausen, Richard-Strauss-Strasse 82, 81679 Munich, Germany; 2 Department of Urology, Ludwig-Maximilians-University of Munich, Marchioninistr. 15, 81377 Munich, Germany

**Keywords:** Acute uncomplicated cystitis (AUC), Resistance profile of *Escherichia coli*, Susceptibility test, Epidemiology

## Abstract

**Background:**

Acute uncomplicated cystitis (AUC) is a common ailment in the urological setting. Guidelines for urinary tract infections are based on large-scale multi-centre, epidemiological and international studies. The objective of this observational study was to establish whether the results of a multi-centre study on the resistance profile of *Escherichia coli (E. coli)* in patients with AUC could be directly applied to an urological practice in a major European city or whether there are divergences in the resistance profile.

**Methods:**

An observational study was applied prospectively to 502 patients with AUC between January 2015 and January 2017). Personal data were anonymised. Exclusion criteria were the patient’s age (<18) and treatment with an antibiotic in the week preceding examination.

**Results:**

The average age was 32 (range 18–56). The most commonly detected bacteria was *E. coli* with 86%, followed by *Enterococcus faecalis* with 10% and *Klebsiella pneumoniae* with 4%. Resistance tests showed *E. coli* to be highly sensitive to fosfomycin (99.2%), nitrofurantoin (98.1%) and cefpodoxime (92.9%). *E. coli* exhibited resistance to ciprofloxacin (CIP) in 15.1%, to trimethoprim/sulfamethoxazole (TRS) in 25.2% and to amoxicillin/clavulanic acid (AMC) in 34% of cases.

**Conclusion:**

The comparison between data from this study and data from a multi-centre European (ECO-SENSI, ECO-SENSII and the 2014 update) showed relatively good sensitivity rates for fosfomycin and nitrofurantoin but significant differences in respect of resistance levels to TRS, CIP and AMC. AUC should therefore only be treated with TRS, CIP and AMC after a susceptibility test has been carried out.

## Background

Uncomplicated acute cystitis is among the most common ailments presented in urological practice. Over recent years the causative bacteria have remained much the same. *Escherichia coli (E. coli)* is still by far the most common uropathogen for acute uncomplicated cystitis (AUC) in women and is found in more than 80% of the positive urine cultures [1]. In practice, the condition is generally treated empirically on the basis of epidemiological studies and resistance testing, although antibiotic treatment is becoming increasingly complex. This is due to the constantly deteriorating resistance profile of *E. coli* strains. This study therefore aimed to determine whether the current recommendations issued by European epidemiological studies [2–6] are directly applicable to a specialist urological practice in a European city and whether the empirical therapy may require adjustment.

## Methods

This prospective study was carried out in a urological practice in a major European city (Munich). From January 2015 to January 2017 a consecutive group of 502 patients with AUC were included in the observational study. Indicators of an AUC were defined in terms of at least one of the following symptoms: suprapubic pain, urge symptoms, dysuria and increased voiding frequency.

Exclusion criteria were if the patient was under the age of 18 or had taken antibiotics during the 7 days prior to examination. The examination medium was a clean catch mid-stream urine sample from the patient. Urine was cultured in the institute’s own laboratory (Instand® certified). This included the identification of the bacteria and resistance testing in accordance with EUCAST criteria. A urine culture was defined as positive if ≥10e3CFU/mL. Mixed cultures were not analysed.

The principles of Section 15 of the Bavarian Rules of Professional Conduct were observed. Consultations were held with the ethics commission of the Bavarian State Chamber of Physicians prior to beginning the study. A separate ethics application was not necessary as the purpose of the research project was quality assurance and imposed neither additional burdens nor examinations on patients and involved no personal data. The criteria of the Declaration of Helsinki were observed. Patients were informed about the project and required to consent in writing. Patients’ data was anonymised once the microbiological results had become available.

## Results

The average age of the patients was 32.7 years (range 18–65). The age peak in the cohort was between ages 18 and 25 (42%). The distribution of urinary tract infections was found to be more common in the age group of 18–25 with 42% and decreased with age to 8–8.2% in women aged 46–65 years. Among pre-menopausal patients AUC was more common than among post-menopausal women (86.9% vs. 13.1%). Three main factors that might influence the rate of AUC in our cohort were recurrent urinary tract infections (20.3%), preceding sexual intercourse (15.3%) and a new sex partner (12.9%). Patient characteristics are shown in Table 1.

**Table 1:** Patient characteristics

Patient characteristics	Absolute	Percentage
	502	100
Age		
Age 18–25	211	42.0
Age 26–35	140	27.9
Age 36–45	70	13.9
Age 46–55	40	8.0
Age 56–65	41	8.2
Average age [range]	32.7 [18–65]	–
Pre-menopause	436	86.9
Post-menopause	66	13.1
Risk factors		
Recurrent urinary tract infections	102	20.3
Diabetes mellitus	15	3.0
Pregnancy	4	0.8
New sex partner	65	12.9
First-degree relative with urinary tract infections	34	6.8
Intercourse-related	77	15.3

After the exclusion of mixed cultures, a total of 423 patients from an initial 502 remained for analysis. The most common bacteria was *E. coli* with 86.3% (365/423) followed by *Enterococcus faecalis* with 10.2% (43/423) and *Klebsiella pneumoniae* with 3.5% (15/423). A cohort of 365 patients was available for *E. coli* analysis. Susceptibility testing showed *E. coli* strains to be highly sensitive to the following oral antibiotics: fosfomycin-trometamol (99.2%), nitrofurantoin (98.1%) and cefpodoxime (92.9%). *E. coli* was resistant to ciprofloxacin in 15.1% of cases, to trimethoprim/sulfamethoxazole in 25.2% of cases and to amoxicillin/clavulanic acid in 25.2% of cases (Table 2).

**Table 2:** Percentage susceptibility of *E. coli* isolated from women with AUC

Antibiotic	Susceptibility of the antibiotic to *E. coli* [in %]
Ampicillin	60.3
Ampicillin/sulbactam	65.5
Amoxicillin	69.6
Amoxicillin/clavulanic acid	74.8
Piperacillin	69.6
Piperacillin/tazobactam	91.8
Cefuroxime	90.7
Cefpodoxime	92.9
Cefotaxime	95.6
Ceftazidime	95.3
Imipenem	100
Meropenem	100
Ciprofloxacin	84.9
Levofloxacin	86.3
Moxifloxacin	86.0
Gentamycin	94.0
Tetracycline	74.8
Nitrofurantoin	98.1
Trimethoprim/sulfamethoxazole	74.8
Fosfomycin-trometamol	99.2

## Discussion

In this prospective observational study we were able to raise “real-world” data on causative pathogens of AUC and their corresponding resistance profiles to antibiotics in an urological practice. Treatment is usually based on empirical data collected from multi-centre studies. It is known that the resistance profiles of causative bacteria changes over the course of time. Not only are these changes time-related, they also differ considerably across regions and nations [3, 5–9]. The question therefore arises whether guideline recommendations for the use of specific antibiotics in AUC are directly applicable to an urological practice in a major German city, or whether such guideline recommendations require regional and national modification.

### Comparison of our data to international studies

Successful empirical treatment is based on the fact that an absolute majority of the pathogenic bacteria actually is susceptible to the selected substance. Unfortunately, experience has shown that resistance rates constantly change adversely. For example, the ECO-SENS I Study (multicentric, European) carried out between 1999 and 2000 and the ECO-SENS II Study (2007–2008) show a shift in resistence rates of *E. coli* to antibiotics in Europa over an 8-year period [5, 6]. A recently published update-2014 of ECO-SENS and simultaneous comparison with the ECO-SENS I and II studies gives alarming resistance rates for France, Germany, Spain, Sweden and UK with regard to ciprofloxacin (4.8% and 30.8%) and to trimethoprim (26.9% and 46.0%) [6]. In addision, our data from this manuscript demonstrate alarming resistence rates for aminopenicillins (ampicillin, amoxicillin in combination with sulbactam and clavulanic acid, respectively) between 25.2 and 39.7% and therefore differs significantly from data in ECO-SENS and the 2014-update (between 4.5 and 28%) [5, 6]. This also applies to ciprofloxacin, ECO-SENS II gives international resistence rates of 3.9%, whereas our study finds this to be 15.1%. Ultimately, it is apparent that resistance rates from a European multi-centre study cannot be randomly applied to a small geographic region in Europe. But on the basis of our data and international studies (ECO-SENS) it can currently be confirmed that fosfomycin-trometamol and nitrofurantoin can be given in our urological practice as first-line therapy without susceptibility testing.

### Comparison of our data to national/regional data

A comparison of *E. coli* susceptibility testing reveals that ECO-SENS I and II contain no data specific to Germany [5, 7]. With regard to our data, the update-2014 (to the studies ECO-SENS I and II) published in 2015 only allows comparisons with nitrofurantoin, ciprofloxacin and amoxicillin/clavulanic acid (fosfomycin was not included in the mentioned study; we did not test the monotherapies mecilliam, cefadroxil and trimethoprim in our study) [6]. In Germany in 2014, resistance rate was found to be 8.3% for amoxicillin/clavulanic acid, 2.3% for nitrofurantoin and 20.3% for ciprofloxacin, the corresponding figures in our study were 25.2, 1.9 and 15.1% respectively. An analysis of the results of the international ARESC Study (Antimicrobial Resistance Epidemiological Survey on Cystitis) on the prevalence and resistance rates of pathogens that cause AUC, particularly in respect of the figures for Germany and our figures, show similarly resistence rates for *E. coli* to ampicillin 40.8% vs. 39.7%, for cefuroxime 8.7% vs. 9.3%, for trimethoprim/sulfamethoxazole 26.0% vs. 25.2%, for nitrofurantoin 4.6% vs. 1.9%, and for fosfomycin 2.1% vs. 0.8%. Significant inconsistencies are found for ciprofloxacin (ARESC 4.6% vs. 15.1%) and amoxicillin/clavulanic acid (ARESC 11.2% vs. 25.2%) [8]. A comparison of our data with the german-wide data shows a pleasant consistency with regard to susceptibility testing for *E. coli* particularly in respect of the antibiotics from the german and european guideline recommendations (fosfomycin and nitrofurantoin) [10]. Our data allows the conclusion that, if treatment with guideline-compliant fosfomycin and nitrofurantoin is not intended, ciprofloxacin, trimethoprim/sulfamethoxazole and especially aminopenicillins should only be prescribed after susceptibility testing.

The reasons for the relative high rate of resistances to ciprofloxacin and amoxicillin/clavulanic acid found in our study is a matter of speculation. A possible reason for an increased resistance situation could be the location of the urological practice in a metropolitan area with its high-density healthcare system and associated high rate of prescription of antibiotics (CIP and AMC). Such antibiotics are not only prescribed for urinary tract infections but also for numerous other medical conditions. On the other hand, there is no plausible explanation for the constantly high level of sensitivity to fosfomycin. It could be equally due to doctors’ restrictive prescription practice and the narrow range of indications for fosfomycin approval. In contrast, a study by Oreo et al. showed that the broader use of fosfomycin in parts of Spain has already caused an increase in resistance rates [11].

The strength of the study lies in its collection of real-world data from a relatively high number of patients in everyday situations in an urological practice. To our knowledge, this is the first publication comparing data from a large cohort in a single centre with national and international multi-centric data. At the same time, the strength of the study is also its weak point. The prospective, single-centre study design does not permit conclusions to be drawn for other parts of the country or Europe, which is absolutely not the study aim. A further disadvantage of the study design could be the 2-year period required to recruit patients. This constitutes a possible bias, as the resistance situation during recruitment could have changed or deteriorated. This, however, does not affect the study findings in general.

In respect of the international and national differences in the resistance profile, for the future it needs to be discussed whether large laboratories in the healthcare system should regularly collect data on the resistance profile of uropathogenic bacteria at national and/or regional level. This could ensure that, in future, empirical AUC therapy can also be initiated without requiring a urine culture and susceptibility testing in each individual case. In the update to the EAU (European Association of Urology) guidelines from March 2015, fosfomycin, pivmecilliam and nitrofurantoin are considered to be first-line therapy [12] with high levels of evidence (LE: 1a, GR: A). In particular fosfomycin exhibits a high level of susceptibility to ESBL-producing *E. coli*, which is already on the decline on some regions of Spain. Furthermore, resistance rates of <20% already suffice to class an antibiotic as first-line medication [13, 14]. This definition renders empirical treatment with aminopenicillins with or without sulbactam or clavulanic acid impossible in our (regional) practice, whereas aminopenicillins could still be used at national level. In this respect, with a resistance rate of 15.1% for ciprofloxacin, it would only be a question of time before empirical therapy with this agent at regional level would no longer make sense.

## Conclusions

Despite the evidence for international and national differences in the resistance profile of *E. coli* to antibiotics, the guidelines can still be applied to empirical treatment of AUC. As significant changes may still occur in regional susceptibility to recommended alternative antibiotics such as ciprofloxacin, aminopenicillins and trimethoprim/sulfamethoxazole, a urine culture with subsequent susceptibility test should be performed before use. Ongoing regular monitoring of the resistance profile at international, national and regional levels could increase the understanding of and reduce reaction times to the development of resistances.
